# Prevalence of sexually transmitted infections and human papillomavirus in cervical samples from incarcerated women in São Paulo, Brazil: a retrospective single-center study

**DOI:** 10.3389/fpubh.2024.1353845

**Published:** 2024-07-23

**Authors:** Marco A. Zonta, Anne Liljander, Karina B. Roque, Arne Schillert, Marco Kai, Flávia A. dos Santo, Giulia Pinheiro de Freitas, Michel Soane, Markus Cavalar, Gustavo Janaudis, Marina Tiemi Shio

**Affiliations:** ^1^Inside Diagnosis, Research and Development S.A., São Paulo, Brazil; ^2^Institute of Experimental Immunology, Affiliated to EUROIMMUN Medizinische Labordiagnostika AG, Lübeck, Germany; ^3^Post Graduation Program in Health Sciences, Santo Amaro University, São Paulo, Brazil; ^4^EUROIMMUN Brasil – EUROInstitute, São Caetano do Sul, Brazil

**Keywords:** sexually transmitted infections, HPV, epidemiology, women’s health, prison, Brazil

## Abstract

**Introduction:**

Sexually transmitted infections (STIs) cause considerable morbidity worldwide and, depending on the specific pathogen, may lead to serious complications in the female reproductive tract. Incarcerated women are particularly vulnerable to health problems with a disproportionate high rate of STIs, including infections with human papillomavirus (HPV).

**Methods:**

Here, cervical swab samples collected from 299 women (18 to 64 years) living in one of the women’s prisons of São Paulo, Brazil were submitted for liquid-based cytology to determine the prevalence of precancerous lesions. Furthermore, direct detection of 30 genital HPV genotypes (18 high-risk and 12 low-risk types) and 11 additional STIs (*Chlamydia trachomatis*, *Neisseria gonorrhoeae*, Herpes simplex virus 1 and 2, *Haemophilus ducreyi*, *Mycoplasma genitalium* and *hominis*, *Treponema pallidum*, *Trichomonas vaginalis*, *Ureaplasma parvum* and *urealyticum*) were performed by molecular typing using two PCR-based DNA microarray systems, i.e., EUROArray HPV and EUROArray STI (EUROIMMUN), respectively.

**Results:**

The overall prevalence of cytological abnormalities was 5.8%, including five women with low-grade and five women with high-grade squamous intraepithelial lesions. The overall prevalence of HPV was 62.2, and 87.1% of the HPV-positive women were infected with oncogenic high-risk (HR) HPV types. HPV types 16 (24.1%), 33 and 52 (both 10.4%) were the most frequently detected. The prevalence of the other STIs was 72.8%. Up to four different pathogens were found in the infected women, the most frequent being *Ureaplasma parvum* (45.3%), *Mycoplasma hominis* (36.2%) and *Trichomonas vaginalis* (24.8%).

**Conclusion:**

The high number of HR-HPV infections and other STIs described here highlights the fact that the Brazilian female prison population requires more attention in the country’s health policies. The implementation of screening programs and treatment measures might contribute to a decrease in the incidence of STIs and cervical cancer in this vulnerable population. However, for such measures to be effective, further studies are needed to investigate the best practice to get more women to engage in in-prison prevention programs, e.g., through offering further sexual health education and self-sampling.

## 1 Introduction

Sexually transmitted infections (STIs) are among the most common acute conditions causing considerable morbidity worldwide. The disease prevalence is particularly high in sexually active women of childbearing age residing in low- and middle-income countries (1), where access to testing and disease management is often limited. Many bacterial STIs such as gonorrhea (*Neisseria gonorrhoeae*), chlamydia (*Chlamydia trachomatis*), and syphilis (*Treponema pallidum* subsp. *pallidum*), as well as viral infections with, e. g., human papillomavirus (HPV), are often asymptomatic and can therefore go undetected for long time periods. If left untreated, some STIs can lead to serious consequences in the female reproductive tract, including the development of pelvic inflammatory disease (PID), ectopic pregnancy and tubal infertility (2–4). Concurrent infections with sexually transmitted pathogens such as parasitic *Trichomonas vaginalis* have also been associated with an increased risk of sexual acquisition and subsequent transmission of HIV (5). Furthermore, prenatal infections with *Ureaplasma* spp., *N. gonorrhoeae* and *C. trachomatis* can induce complications such as miscarriage, premature labour, low birth weight and neonatal pneumonia (6–8). Moreover, persistent infections with oncogenic high-risk (HR) human papilloma virus (HPV) types have been associated with the development of precancerous lesions and cervical malignancies, with viral detection in 95% of the cervical cancer cases worldwide, predominantly HR-HPV types 16, 18, and 35 (9, 10). In addition to behavioural co-factors such as parity, smoking and long-term use of oral contraceptives, co-infections with other STIs have also been associated with the progression from cervical HPV infection to cellular abnormalities and invasive cancer (11, 12).

Cervical cancer screening programs are an efficient tool to reduce the number of disease cases, and similarly, HPV vaccination plays a crucial role. Studies have shown that in middle-income countries like Brazil, vaccinating pre-adolescent girls is cost-effective, especially if high vaccination coverage rates can be achieved (13). HPV vaccination was incorporated into the Brazilian Unified Health System in 2014, where it initially was offered to girls from 9 to 11 years of age free of charge. The current recommendation is gender neutral and includes vaccination of children and adolescent between 9 and 14 years old and immunosuppressed people up to 45 years old (14). However, despite this, cervical cancer consistently remains one of the most frequent cancers in Brazilian women, ranking 4th in 2018 (15). The high incidence and mortality could be partly attributable to regional differences in the coverage of preventive screening (16) as well as vaccination (17).

Due to risk behaviors such as substance and alcohol abuse, multiple sex partners, commercial sex work and unprotected sex, incarcerated women represent a particularly vulnerable population for development of gynecological problems (18–20). Higher rates of STIs, HPV infections, precancerous lesions and cervical cancer have been reported among incarcerated women compared to women in the general population (20, 21). Older adult imprisoned women are also at a substantially higher risk of developing cervical cancer (22). Brazil has one of the largest populations of incarcerated women in the world (23). Only between the years 2000 and 2016 the number of imprisoned women increased by over 650% (24). This is much due to harshening penalties for small-scale drug trafficking, a task often performed by women to earn some income for their livelihood and that of their families. Women are often the main or only provider for their children and approximately 60% of the incarcerated women are mothers (25). Although incarcerated individuals should have access to health care services of the same standard as available in the community (26), this is unfortunately not always the case in Brazil (24, 27). Furthermore, official epidemiological data regarding notifiable infectious diseases in prison environments are scarce and most published data is focused on tuberculosis and HIV/AIDS (27). Thus, additional studies addressing the prevalence of other diseases e. g., STIs are warranted. One study demonstrated large differences in the access to cervical screening tests and treatment in female penitentiaries across the state of Mato Grosso do Sul, with only approximately 50% of the women reporting having received a Papanicolaou test (Pap test) during their incarceration (28). Furthermore, the prevalence of HPV infection in the female prison population in the city of São Paulo, Brazil was high, as indicated by a 44% prevalence rate of HR-HPV types (HPV 16, 18, 33 and 39) determined by molecular testing (29). In addition, some studies have reported high rates of STIs in women in Brazilian prisons (19, 30, 31). Characterizing the disease burden, both asymptomatic and symptomatic infections in these high risk populations, living under unhygienic conditions, in often overcrowded prisons and with limited access to basic health care (32), is of great importance. Not only for providing adequate treatment, thus improving the sexual and reproductive health of these vulnerable women, but also from a public health perspective by limiting disease transmission inside and outside the prison.

In this study, the prevalence of precancerous lesions, 30 HPV genotypes and 11 additional STIs was determined in cervical swab samples collected from incarcerated women at one of the women’s prisons in São Paulo, Brazil.

## 2 Materials and methods

### 2.1 Study population

This cross-sectional screening was conducted between June 2015 and February 2016 at one of the women’s prisons of the city of São Paulo, Brazil. At the time of the study, the prison health service team consisted of a full-time nurse and a medical doctor visiting the prison once per week. Cytology based screening for cervical cancer has been available (upon request) since 2011 through a women’s health program, initiated by the principal investigator of this study (Dr Zonta). No HPV testing was performed and any additional testing, e.g., for STIs was symptom-based. At the time of the study approximately 2,680 women were living in the correctional facility. Prior to the study, a series of group meetings were held where the women were informed about HPV in general, viral transmission routes and its association with cervical cancer. Newly arriving women were informed in a catch-up meeting held 1 week before the start of the study. All non-pregnant women aged ≥18 years were invited to participate in the screening.

The recruitment and scheduling of the women, the follow-up of collection procedures and organization of the site during the activities was supervised by the principal investigator of this study, while the sample collection was performed and overseen by members of the prison health service team. Written informed consent was obtained from all participants before their inclusion in the study. The women, who accepted to participate were interviewed using a self-administered paper-based questionnaire that included closed-ended questions on sociodemographic factors and sexual behaviour. A member of the research team was available if the women had any inquiries or if they had any difficulties in understanding any of the questions.

The results from the analyses were forwarded to the medical clinic at the prison. If any abnormalities were detected the women were referred to the public hospital for further examinations and treatment if needed.

The study was approved by the Ethics Committee of Santo Amaro University – SP (Plataforma Brasil – CAAE: 61414216.4.0000.0081).

### 2.2 Sampling procedures, cervical cytology, HPV genotyping and STI screening

Cervical material was collected from ecto- and endocervical regions using a standard cytobrush (Kolplast™ C I Ltda, Brazil). The sample brush was conserved in a liquid-based cytology medium (CellPreserv – Kolplast™ C I Ltda, Brazil). All samples were sent to the cytology laboratory at the Santo Amaro University for processing. Cervical cell suspensions were fixed and processed using a KLP 2000 slide processor (Kolplast™ C I Ltda, Brazil) according to the manufacturer’s instructions. Following Papanicolaou (Pap) staining, the slides were analysed and classified according to the 2001 Bethesda system.

The remaining medium was stored at – 20 °C until molecular analyses. DNA was extracted from 1 mL medium using the QIAamp DNA Mini Kit (QIAGEN, Germany) according to the manufacturer’s instructions.

HPV genotyping was performed using a PCR-based DNA microarray system (EUROArray HPV, EUROIMMUN Medizinische Labordiagnostika AG, Germany). This full HPV genotyping system allows for the simultaneous detection and differentiation of 18 high-risk (HR) types (16, 18, 26, 31, 33, 35, 39, 45, 51, 52, 53, 56, 58, 59, 66, 68, 73 and 82) and 12 low-risk (LR) types (6, 11, 40, 42, 43, 44, 54, 61, 70, 72, 81 and 89).

The STI prevalence was determined using a similar DNA microarray system, EUROArray STI (EUROIMMUN, Germany). The array allows for simultaneous detection of 11 pathogens: *Chlamydia trachomatis*, Herpes simplex viruses 1 and 2 (HSV-1 and HSV-2), *Haemophilus ducreyi*, *Mycoplasma genitalium*, *Mycoplasma hominis*, *Neisseria gonorrhoeae*, *Treponema pallidum, Trichomonas vaginalis*, *Ureaplasma parvum* and *Ureaplasma urealyticum*. Both arrays (HPV and STI) were performed and evaluated according to the instructions provided by the manufacturer.

### 2.3 Statistical analysis

Sociodemographic and variables related to sexual activity and history, which are associated with HPV status (pos / neg) or the infection diversity (1 vs. >1 HPV type) were selected using group least absolute shrinkage and selection operator (LASSO) analyses (33, 34). Selected group variables were subsequently included in multivariate logistic regression models. The significance level for all analyses was set as α = 0.05. Comparisons of the STI prevalences between HPV-positive and negative women were performed using Pearson’s Chi-squared test. All statistical analyses were performed using R, version 4.0.2 (35). For the group LASSO analysis, the R package grpreg version 3.3.0 was used (36).

## 3 Results

### 3.1 Characteristics of the participants

In total, 299 incarcerated women agreed to participate in the study and the results are based on data from all women, unless stated otherwise (the number of women answering the individual questions is indicated in brackets). The participants’ ages ranged from 18 to 64 years (mean = 31.8 years). The literacy rate among the 297 responding women was 100%. In total, 79.8% (225 / 282) women reported having a profession. More than half of the women (57.4%, 171 / 298) stated that they were not engaged in a formal relationship (i.e., being single) and most of them (81.9%, 245 / 299) were below the age of 18 at the time of their first sexual intercourse. In total, 57.5% (168 / 292) of the women reported having had between one to five- life-time sexual partners. Moreover, most of the interviewed women reported having been pregnant more than two times (66.2%, 198 / 299). Furthermore, 82.3% (246 / 299) women had children; more than half of them (57.5%, 172 / 299) had one to three children while 24.8% (74 / 299) had four or more children (Table 1).

**Table 1:** Sociodemographic and sexual characteristics of the incarcerated women in São Paulo, Brazil.

Characteristics	HPV (+) *n* = 186 (%)	HPV (−) *n* = 113 (%)	Total *n* = 299 (%)
**Age (years)**			
≤25	54 (29.0)	24 (21.2)	78 (26.1)
26–30	43 (23.1)	30 (26.5)	73 (24.4)
31–44	72 (38.7)	49 (43.4)	121 (40.5)
≥45	17 (9.1)	10 (8.8)	27 (9.0)
Education level*			
Elementary school	87 (47.0)	57 (50.9)	144 (48.5)
High school	72 (38.9)	42 (37.5)	114 (38.4)
University	26 (14.1)	13 (11.6)	39 (13.1)
Profession*			
Unemployed	10 (5.7)	8 (7.5)	18 (6.4)
Student	4 (2.3)	2 (1.9)	6 (2.1)
Professional	148 (84.1)	77 (72.6)	225 (79.8)
Homemaker	14 (8.0)	19 (17.9)	33 (11.7)
Marital status*			
Single	105 (56.8)	66 (58.4)	171 (57.4)
Married	65 (35.1)	28 (24.8)	93 (31.2)
Divorced/widowed	15 (8.1)	19 (16.8)	34 (11.4)
**Age at first sexual intercourse**			
≤18	151 (81.2)	94 (83.2)	245 (81.9)
≥19	35 (18.8)	19 (16.8)	54 (18.1)
No. of lifetime sexual partners*			
1 to 5	96 (51.6)	72 (65.5)	168 (57.5)
6 to 15	45 (24.2)	15 (13.6)	60 (20.6)
≥16	41 (22.0)	23 (20.9)	64 (21.9)
**Pregnancy**			
Never	29 (15.6)	13 (11.5)	42 (14.1)
One time	36 (19.4)	23 (20.4)	59 (19.7)
≥2 times	121 (65.1)	77 (68.1)	198 (66.2)
**No. children**			
0	36 (19.4)	17 (15.0)	53 (17.7)
1 to 3	118 (63.4)	54 (47.8)	172 (57.5)
≥4	32 (17.2)	42 (37.2)	74 (24.8)
**Regular condom use**			
No	104 (55.9)	71 (62.8)	175 (58.5)
Yes	82 (44.1)	42 (37.2)	124 (41.5)
**Sex for money**			
No	141 (75.8)	92 (81.4)	233 (77.9)
Yes	45 (24.2)	21 (18.6)	66 (22.1)
**Sex with women**			
No	118 (63.4)	74 (65.5)	192 (64.2)
Yes	68 (36.6)	39 (34.5)	107 (35.8)
STI history*			
Previous history	39 (21.4)	19 (17.4)	58 (19.9)
No previous history	133 (73.1)	87 (79.8)	220 (75.6)
Do not know	10 (5.5)	3 (2.8)	13 (4.5)
Anal intercourse*			
No	89 (47.8)	65 (58.0)	154 (52.0)
Yes	97 (52.2)	47 (42.0)	144 (48.0)
**Sexual assault**			
No	145 (78.0)	92 (81.4)	237 (79.3)
Yes	41 (22.0)	21 (18.6)	62 (20.7)
**Smoking**			
No	75 (40.3)	45 (39.8)	120 (40.1)
Yes	111 (59.7)	68 (60.2)	179 (59.9)

* Does not add up to 299 since some responses are missing.

Of the interviewed women, 41.5% (124 / 299) reported that they always used condoms when having sex, 22.1% (66 / 299) stated that they have had sex in exchange for money and 35.8% (107 / 299) of the women have had sex with other women. In total, 75.6% (220 / 291) women reported that they have had no previous STIs, 19.9% stated that they have had STIs, while 4.5% did not know their history of infection. Nearly half (48.0%, 144 / 298) of the responding women reported having had anal sex. One out of five women (20.7%, 62 / 299) reported having been sexually assaulted. In total, 59.9% (179 / 299) of the women used tobacco (Table 1). A low percentage of the women consumed alcohol (2.3%) and none of the women were vaccinated against HPV.

### 3.2 Cytological examination

In the 277 cervical smear samples considered satisfactory for cytopathological examination, the overall prevalence of precancerous lesions was 5.8% (16 / 277), including six samples (2.2%) with atypical squamous cells of undetermined significance (ASC-US), five samples (1.8%) with low-grade squamous intraepithelial lesions (LSIL) and five samples (1.8%) with high-grade squamous intraepithelial lesions (HSIL). The age of the women presenting with precancerous lesions ranged between 20 and 49 years (mean 29.8 years). Of the 261 / 277 samples (94.2%) that were negative for cytological abnormalities, 32 were classified as normal, i.e., showed no reactive alterations in cellular morphology, while the rest (*n* = 229) were classified as reactive, compatible with cervical inflammation.

### 3.3 HPV infection prevalence and genotype

**Figure 1:**
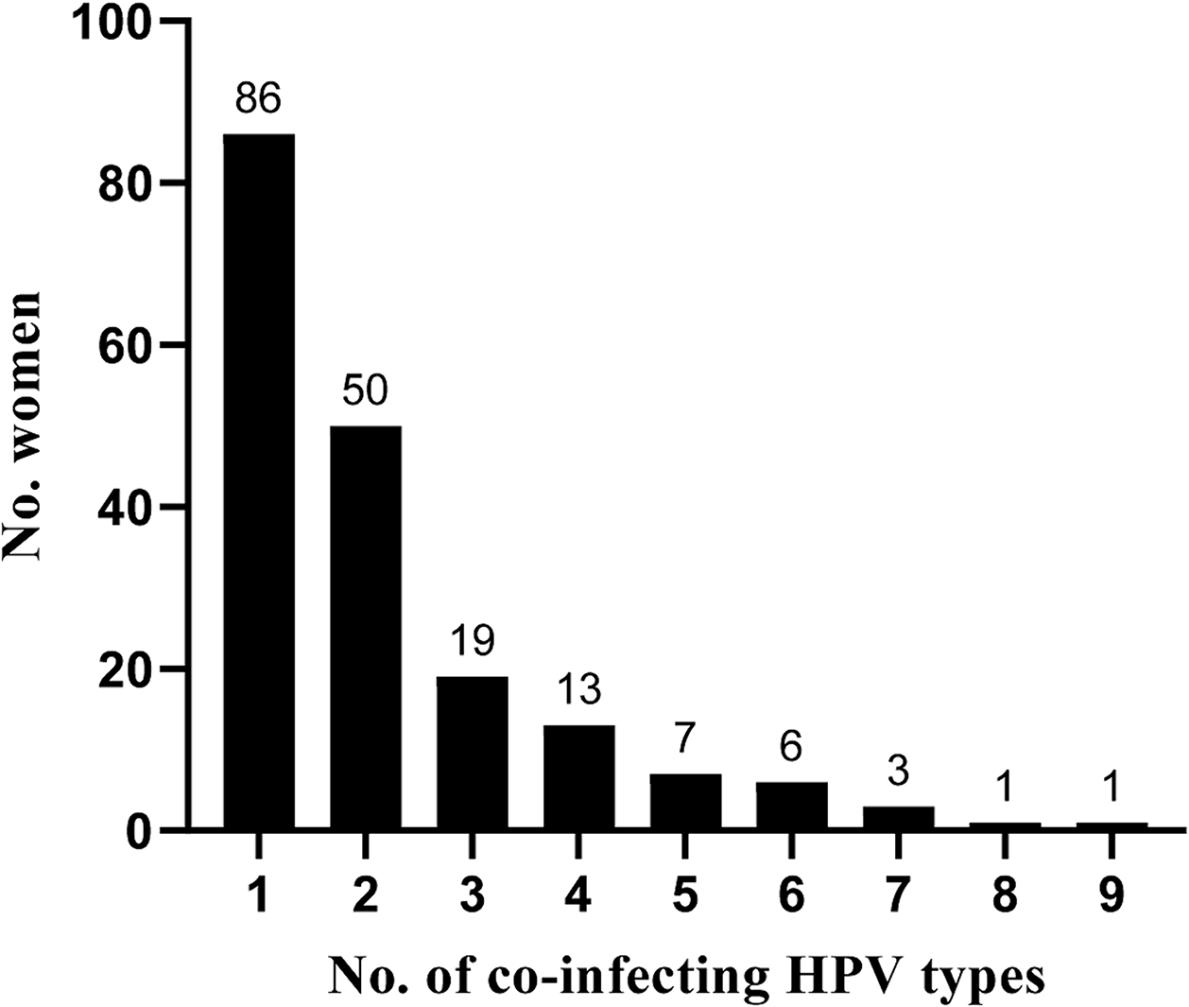
Number of co-infecting HPV types among the incarcerated women in São Paulo, Brazil.

**Figure 2:**
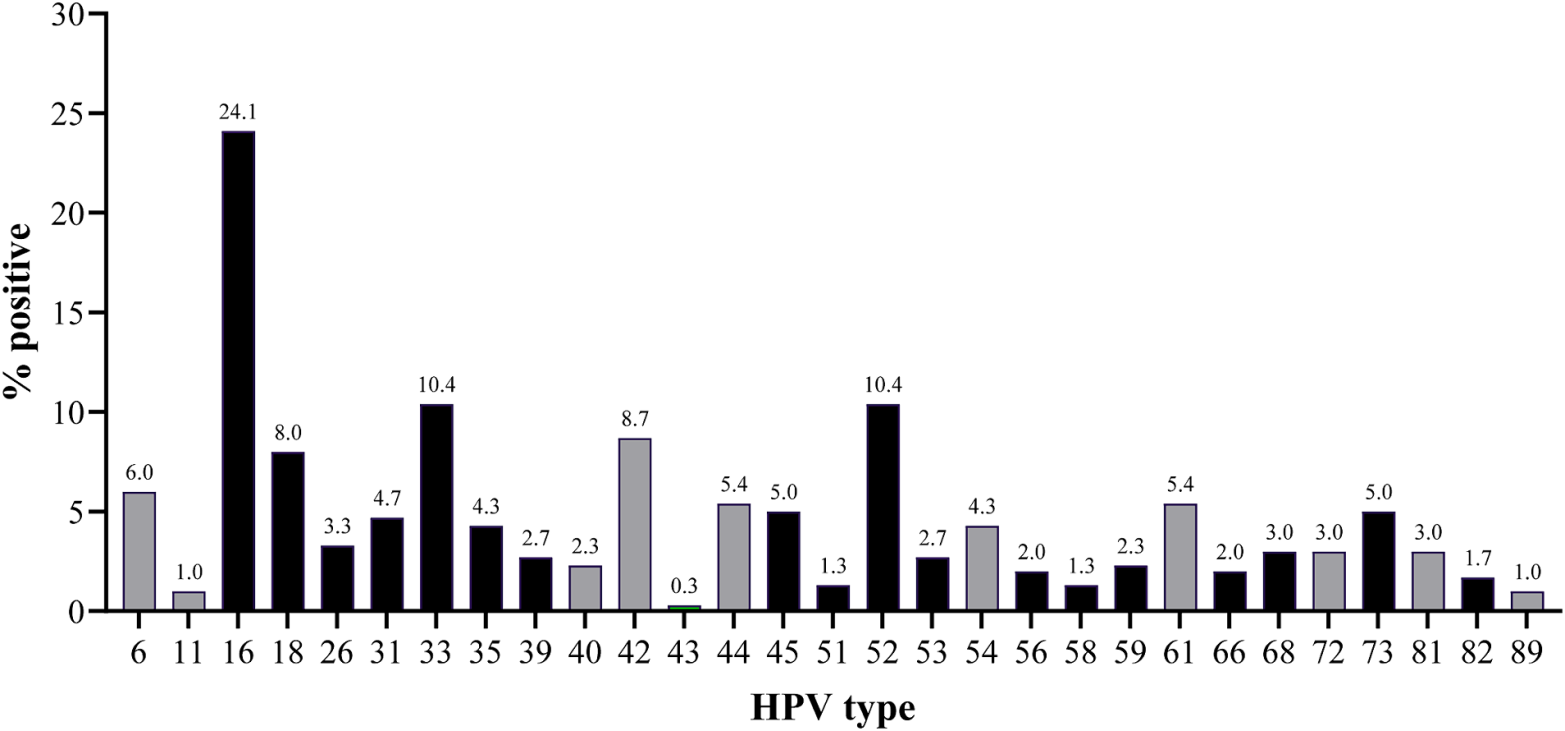
Frequency of the different HPV types in the incarcerated women in São Paulo, Brazil. HR-HPV and LR-HPV types are indicated in black and grey, respectively.

Of the investigated women, 62.2% (186 / 299) were positive for any HPV type. The overall prevalence of HR-HPV types, either as single infection or in combination with other HR types or with LR-HPV types, was 54.2% (162/299). Among the women that tested positive for HPV, 53.8% (100 / 186) were infected with two or more HPV types (range 2 to 9 types, Figure 1). In total, 29 different types were detected, with HPV 16 (24.1%), HPV 33 (10.4%), HPV 52 (10.4%), HPV 42 (8.7%) and HPV 18 (8.0%) being the most prevalent types (Figure 2). Seven women were infected with both HPV 16 and 18. Of the 16 women presenting with any precancerous lesion (ASC-US, LSIL or HSIL), 14 had detectable HPV infections. Of these women, 11 were infected with HR-HPV types (71.4%), while all women presenting with LSIL or HSIL were infected with HR-HPV types (Table 2). The prevalence of multiple infections in the latter group was 77.8%. One woman with ASC-US and one woman with HSIL had no detectable HPV infection.

**Table 2:** Frequency of the different HPV types in the incarcerated women in São Paulo, Brazil.

Cytopathology	HR-HPV	LR-HPV
ASC-US	26, 45	−
ASC-US	−	6, 44
ASC-US	16, 31, 39	44, 81
ASC-US	−	6
ASC-US	−	54
ASC-US	−	−
LSIL	68	72
LSIL	18, 45	42
LSIL	16	72
LSIL	33, 52, 53,	6, 81
LSIL	26	−
HSIL	52	−
HSIL	16, 31, 51	6, 44
HSIL	18, 45	−
HSIL	16, 31	−
HSIL	−	−

ASC-US, atypical squamous cells of undetermined significance; LSIL, low-grade squamous intraepithelial lesions; HSIL, high-grade squamous intraepithelial lesions; −, negative.

### 3.4 Prevalence of sexually transmitted infections

**Figure 3:**
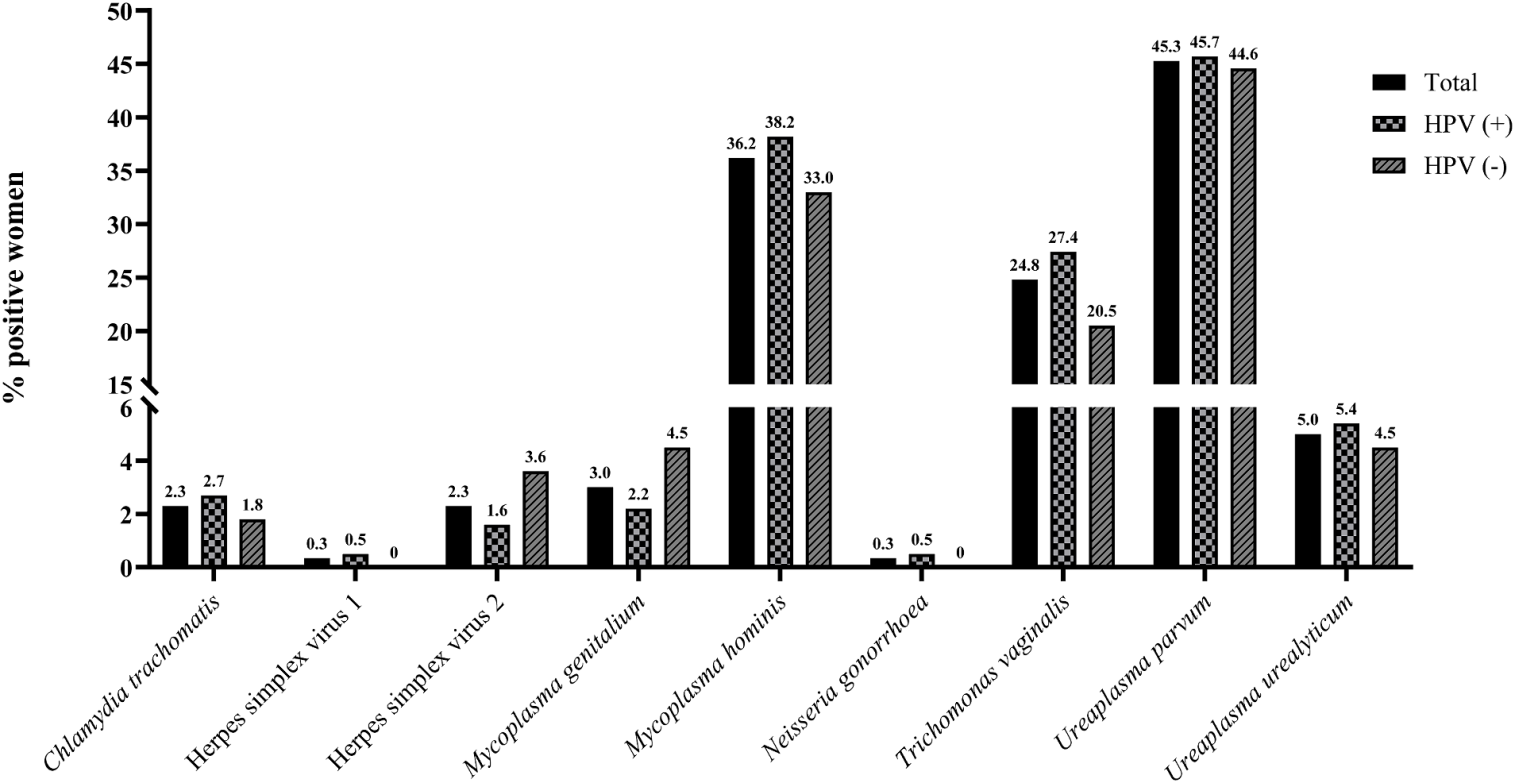
Prevalence of STIs among the imprisoned women. The y-axis has been segmented in order to display all results. STIs for which all women were negative for have been excluded.

In total 217 / 298 (72.8%) women were positive for one or more STIs. One woman had an invalid result in the testing and was excluded from the analysis. Of the positive women, a comparable percentage was infected with one (51.6%) or at least two infecting microorganisms (48.4%). *U. parvum* and *M. hominis* were the most frequently detected infective agents with 45.3% (135/298) and 36.2% (108/298) of the women being infected, respectively. *T. vaginalis* was identified in 24.8% (74/298) of the women. The prevalence of the other eight STIs was low (0 to 5%) (Figure 3; Supplementary Table S1).

### 3.5 Factors associated with HPV status and cytopathology

Using a group LASSO analysis, six variables were identified as key determinants for the HPV status (pos /neg): profession, number of pregnancies and number of children, current STI status (pos /neg), condom use and sexual assault. However, none of the factors were significantly associated with the HPV status in the multivariate logistic regression model (Table 3). A similar LASSO analysis identified the following determinants of infection diversity (1 vs. > 1 infecting HPV type): age category, profession, marital status, age at first sexual intercourse, number of lifetime sexual partners, condom use and sexual assault. However, similarly to the HPV status, no individual variables were significantly associated with infection diversity (Supplementary Table S2).

**Table 3:** Multivariate analysis of factors associated with HPV status among the incarcerated women in São Paulo, Brazil.

Characteristics	OR	95% CI	*p*
**Profession**			
Unemployed	1		
Student	2.67	0.29–60.46	0.43
Professional	1.34	0.44–3.96	0.60
Homemaker	0.59	0.15–2.19	0.43
**Pregnancy**			
Never	1		
One time	0.71	0.15–3.99	0.67
≥2 times	1.17	0.23–7.07	0.85
**No. children**			
0	1		
1 to 3	1.03	0.20–4.22	0.96
≥4	0.27	0.05–1.21	0.10
**Regular condom use**			
No	1		
Yes	0.91	0.52–1.59	0.75
**Sexual assault**			
No	1		
Yes	1.84	0.94–3.74	0.08

OR, odds ratio; CI, confidence interval.

Notably, infections with *M. hominis* were more frequent in HPV-positive than HPV-negative women, 38.2% (71/186) vs. 33.0% (37/112), while infections with HSV-2 were more frequent in HPV-negative women (Figure 3; Supplementary Table S1). Furthermore, *T. vaginalis* infections were more common in women that were positive for HPV (27.4% [51/186] vs. 20.5% [23/112]) and in women presenting with cervical abnormalities (ASC-US, LSIL and HSIL) compared to the other women (50.0% [8/16] vs. 23.1% [65/282]). However, the differences were not significant (Supplementary Table S1).

## 4 Discussion

Incarcerated women are a group that is particularly vulnerable to health problems, with a disproportionate high rate of infectious diseases, in particular sexually transmitted infections (STIs) such as HPV (21). This fact may be related to risk factors occurring prior to imprisonment, such as low socioeconomic status and high-risk sexual behaviour, but may also be due to unprotected sexual activities and limited access to healthcare and prevention measures during incarceration. Besides increasing the risk of gynecological conditions such as PID, infertility and cervical cancer (21), untreated STIs might also be transmitted to susceptible partners. Therefore, targeted measures of STI screening and treatment in prisons can help to improve women prisoner’s health and stop the chain of disease transmission. In fact, previous studies have demonstrated that correctional facilities are important sites for STI screening and treatment implementation (37, 38) and in addition to *T. pallidum* testing for pregnant women (39), routine screening of female detainees for, e.g., *C. trachomatis*, *T. vaginalis* and *N. gonorrhoeae* has been recommended (40, 41). Besides improving the wellbeing of the individual, such measures might have positive impact on the community prevalence of STIs (42). Assessing the prevalence of HPV and other STIs in these high-risk populations is pivotal for such implementation programs. Here, the prevalence of precancerous lesions, HPV and 11 other STIs was investigated in a group of incarcerated women in one of the women’s prisons of the city of São Paulo, Brazil. In this study, 5.8% of the women presented with cytological abnormalities. Furthermore, 62.2% of the women were positive for any HPV type.

The HPV prevalence was higher than that recently reported in a meta-analysis of studies in Brazilian women with and without cytology, which showed an overall prevalence of 25.4% (43). It is also substantially higher than what has been described in studies investigating women in São Paulo (20.2 and 48.6% HPV positive) and women with normal cytology in Brazil (6.7% HPV positive) (44).

Higher HPV prevalence in imprisoned women has been described previously (21). The overall prevalence of HR-HPV types was 54.2%, corresponding to a percentage of 87.1 in HPV-positive women only. These frequencies are substantially higher than those reported for the general female population (17.7%) in Brazil (43). Such high HPV and HR-HPV prevalences have also been described in other risk groups, such as women with HIV (45) and commercial sex workers (46). It should be noted that the HIV status of the women investigated in this study is not known. The high prevalence of HPV, in particular HR-HPV types detected in this study, highlights the importance of offering tailored screening programs in correctional institutions in order to ensure access to testing and, if needed, medical treatment. Furthermore, incarceration could also present a unique opportunity to offer HPV vaccination of eligible women. While the attitudes and barriers to receiving in-prison vaccinations were not investigated here, previous studies have shown a willingness among the female inmates to receive immunizations during incarceration (47, 48).

Extended HPV genotyping is an important tool for risk determination in cervical cancer screening programs as well as verifying the effectiveness of vaccination programs against infection (49–51). Here, a DNA array enabling simultaneous differentiation of 30 HPV types was used. All types but one (HPV 70) were detected, with HPV 16 (24.1%), HPV 33 (10.4%), HPV 52 (10.4%), HPV 42 (8.7%) and HPV 18 (8.0%) being the most frequently detected (Figure 2). Except for HPV 42, all these types are HR-HPV types and are associated with cumulative risks for precancerous lesions by year seven, ranging between >20% for HPV 16 and > 5% for HPV 33 and HPV 52 (50). The HPV types reported here were also the most frequently found types in Brazilian women with and without cytology (52).

Infections with multiple HPV types were detected in 53.8% of the HPV-positive women and in 77.8% of the women presenting with precancerous lesions (LSIL and HSIL). The role of HPV co-infections in the development and progression of such lesions and cervical cancer is unclear. Previous studies have suggested that concurrent infections with multiple HPV types are associated with an increased risk of precancerous lesions (53–55), while other studies have found no increased risk associated with multiple infections (56). The risk of cervical cancer or its precursors is, however, elevated in women with other co-infecting agents such as *C. trachomatis*, HSV and *T. vaginalis* (11, 57). Likewise, cervical inflammation has been suggested to be a contributing factor to HSIL (58). Of the women investigated here, 82.7% showed signs of inflammation in their cervical samples. Furthermore, 72.8% were infected with one or more STIs. *U. parvum* and *M. hominis* were commonly detected. Both bacteria frequently occur in the female lower genital tract and are associated with HPV infections (59, 60). Here, a higher prevalence was also found in HPV-positive women. The difference between HPV-positive and -negative women was, however, not significant.

Similarly, to what has previously been reported (57), the prevalence of *T. vaginalis* was higher in HPV-positive women (27.4% vs. 20.5%), in particular in women displaying cervical abnormalities (50.0% vs. 23.1%). However, in contrast to what was reported previously (59, 61), infections with HSV-2 and *M. genitalium* were more frequent in HPV-negative women (Figure 3; Supplementary Table S1). The difference between HPV-positive and -negative women was, however, not significant. Furthermore, although many women with risk profiles, i.e., many sexual partners (in total 42.5% had more than 6 lifetime partners), low age at first sex (81.9%), numerous pregnancies and several children, were included, no significant associations were found between the HPV status and the sociodemographic factors identified as key determinants in the group LASSO analysis (Table 3).

### 4.1 Study limitation

Sample size was considered a limitation of this study. The authors can only speculate regarding the reasons why so few women were willing to participate. However, intimate gynecological examinations have the potential for causing embarrassment, anxiety, and discomfort, making women reluctant. For future studies, vaginal self-sampling should be offered to overcome the emotional and practical barriers to potentially increase the participation in cervical screening. The authors therefore acknowledge that the results presented here represent a small group of the sexually active incarcerated women in Brazil. Furthermore, the questionnaire used in this study exhibited limitations in its level of detail, which hindered the identification of statistically significant factors associated with HPV status. In addition, although symptomatic and asymptomatic viral shedding is common for both HSV-1 and HSV-2, the prevalence of these infections is likely to have been underestimated when only applying direct detection and not serology.

## 5 Conclusion

There has been a sharp increase in the incarceration rate of females in Brazil between 2000 and 2016, mainly driven by a harshening in the sentencing for minor drug-trafficking offences. Since women in prison are more likely to engage in risk behaviors such as having multiple sex partners, commercial sex work and unprotected sex, they are particularly susceptible to gynecological problems. Furthermore, over-crowding and a lack of basic healthcare contributes to their already precarious situation. Imprisonment presents a distinct opportunity to offer preventive services that these at-risk women might otherwise lack access to. Health measures that could be of particular importance to incarcerated women include cervical cancer screening and infectious disease testing and immunization (STIs). The data generated here demonstrates that the burden of infectious diseases, i.e., HPV and other STIs is high among incarcerated women in São Paulo, Brazil. Moreover, not only are these infections often asymptomatic, but also transmissible, further highlighting the important public health role that a prison could serve in the detection and management of these diseases. Given the right support, prisons could potentially also provide HPV vaccinations to eligible women, thus further reducing the morbidity and mortality from cervical and other HPV-related cancers. However, for such measures to be effective, further studies are needed to investigate the best practice to get more women to engage in in-prison prevention programs, e.g., through offering further sexual health education and self-sampling.

## Data availability statement

The raw data supporting the conclusions of this article will be made available by the authors, without undue reservation.

## Ethics statement

The studies involving humans were approved by Ethics Committee of Santo Amaro University – SP (Plataforma Brasil – CAAE: 61414216.4.0000.0081). The studies were conducted in accordance with the local legislation and institutional requirements. The participants provided their written informed consent to participate in this study.

## Author contributions

MZ: Conceptualization, Formal analysis, Investigation, Supervision, Writing – original draft, Writing – review & editing. AL: Data curation, Formal analysis, Visualization, Writing – original draft, Writing – review & editing. KR: Formal analysis, Writing – review & editing. AS: Data curation, Formal analysis, Writing – review & editing. MK: Formal analysis, Writing – original draft, Writing – review & editing. FS: Data curation, Writing – review & editing. GF: Data curation, Resources, Writing – review & editing. MiS: Data curation, Writing – review & editing. MC: Resources, Writing – review & editing. GJ: Resources, Writing – review & editing. MaS: Data curation, Investigation, Visualization, Writing – original draft, Writing – review & editing.
