# Supplementary material for: Prevalence of sexually transmitted infections and human papillomavirus in cervical samples from incarcerated women in São Paulo, Brazil: a retrospective single-center study
Source: Front Public Health. 2024 Jul 23;12:1353845. doi: 10.3389/fpubh.2024.1353845 (PMC11300339; doi:10.3389/fpubh.2024.1353845)
Supplement: Supplementary file 1 [file Table_1.docx]

**SUPPLEMENTARY TABLE 1.** Prevalence of sexually transmitted infections among the incarcerated women in São Paulo, Brazil. (* One woman with a non-valid result has been excluded from the analysis, **^#^** Pearson’s Chi-squared test)

| **Microorganism**  positive (%) | **Total**  **n=298* (%)** | **HPV (+)**  **n=186 (%)** | **HPV (-)**  **n=112* (%)** | **p^#^** |
| --- | --- | --- | --- | --- |
| *Chlamydia trachomatis* | 7 (2.3) | 5 (2.7) | 2 (1.8) | 0.611 |
| *Haemophilus ducreyi* | 0 (0) | 0 (0) | 0 (0) | - |
| Herpes simplex virus 1 | 1 (0.34) | 1 (0.5) | 0 (0) | 0.435 |
| Herpes simplex virus 2 | 7 (2.3) | 3 (1.6) | 4 (3.6) | 0.285 |
| *Mycoplasma genitalium* | 9 (3.0) | 4 (2.2) | 5 (4.5) | 0.264 |
| *Mycoplasma hominis* | 108 (36.2) | 71 (38.2) | 37 (33.0) | 0.343 |
| *Neisseria gonorrhoea* | 1 (0.34) | 1 (0.5) | 0 (0) | 0.435 |
| *Treponema pallidum* | 0 (0) | 0 (0) | 0 (0) | - |
| *Trichomonas vaginalis* | 74 (24.8) | 51 (27.4) | 23 (20.5) | 0.170 |
| *Ureaplasma parvum* | 135 (45.3) | 85 (45.7) | 50 (44.6) | 0.807 |
| *Ureaplasma urealyticum* | 15 (5.0) | 10 (5.4) | 5 (4.5) | 0.715 |
