# Supplementary material for: Prevalence of sexually transmitted infections and human papillomavirus in cervical samples from incarcerated women in São Paulo, Brazil: a retrospective single-center study
Source: Front Public Health. 2024 Jul 23;12:1353845. doi: 10.3389/fpubh.2024.1353845 (PMC11300339; doi:10.3389/fpubh.2024.1353845)
Supplement: Supplementary file 2 [file Table_2.docx]

**SUPPLEMENTARY TABLE 2.** Factors associated with HPV infection diversity (1 vs. >1 HPV types) in the incarcerated women in São Paulo, Brazil.

| **Characteristics** | **OR** | **95 % CI** | **p** |
| --- | --- | --- | --- |
| **Age (years)** | | | |
| ≤25 | 0.78 | (0.20-2.96) | 0.71 |
| 26-30 | 0.55 | (0.14-2.13) | 0.38 |
| 31-44 | 0.68 | (0.19-2.35) | 0.53 |
| ≥45 | 1 |  |  |
| **Profession** | | | |
| Unemployed | 1 |  |  |
| Student | 1.44 | (0.11-38.92) | 0.79 |
| Professional | 0.41 | (0.09-1.74) | 0.22 |
| Homemaker | 0.88 | (0.14-5.30) | 0.89 |
| **Marital status** | | | |
| Single | 1 |  |  |
| Married | 0.59 | (0.28-1.21) | 0.15 |
| Divorced/widowed | 0.43 | (0.10-1.55) | 0.21 |
| **Age at first sexual intercourse** | | | |
| ≤18 | 1 |  |  |
| ≥19 | 2.24 | (0.94-5.49) | 0.07 |
| **No. of lifetime sexual partners** | | | |
| 1 to 5 | 1 |  |  |
| 6 to 15 | 1.33 | (0.58-3.04) | 0.50 |
| ≥16 | 0.85 | (0.36-1.98) | 0.70 |
| **Regular condom use** | | | |
| No | 1 |  |  |
| Yes | 2.03 | (1.02-4.09) | 0.05 |
| **Sexual assault** | | | |
| No | 1 |  |  |
| Yes | 1.43 | (0.64-3.17) | 0.38 |
